# Prospective observational study of vaginal microbiota pre‐ and post‐rescue cervical cerclage

**DOI:** 10.1111/1471-0528.15600

**Published:** 2019-03-10

**Authors:** RG Brown, D Chan, V Terzidou, YS Lee, A Smith, JR Marchesi, DA MacIntyre, PR Bennett

**Affiliations:** ^1^ Queen Charlotte's & Chelsea Hospital Imperial College Healthcare National Health Service Trust London UK; ^2^ Imperial College Parturition Research Group Division of the Institute of Reproductive and Developmental Biology Imperial College London London UK; ^3^ Chelsea & Westminster Hospital Chelsea & Westminster National Health Service Foundation Trust London UK; ^4^ School of Biosciences Cardiff University Cardiff UK; ^5^ Centre for Digestive and Gut Health Imperial College London London UK

**Keywords:** Infection, preterm birth, rescue cerclage, vaginal microbiome

## Abstract

**Objective:**

To investigate the relation between vaginal microbiota composition and outcome of rescue cervical cerclage.

**Design:**

Prospective observational study.

**Setting:**

Queen Charlotte's and Chelsea Hospital, London.

**Population:**

Twenty singleton pregnancies undergoing a rescue cervical cerclage.

**Methods:**

Vaginal microbiota composition was analysed in women presenting with a dilated cervix and exposed fetal membranes before and 10 days following rescue cervical cerclage and was correlated with clinical outcomes.

**Main outcome measures:**

Composition of vaginal bacteria was characterised by culture‐independent next generation sequencing. Successful cerclage was defined as that resulting in the birth of a neonate discharged from hospital without morbidity. Unsuccessful cerclage was defined as procedures culminating in miscarriage, intrauterine death, neonatal death or significant neonatal morbidity.

**Results:**

Reduced *Lactobacillus* spp. relative abundance was observed in 40% of cases prior to rescue cerclage compared with 10% of gestation age‐matched controls (8/20, 40% versus 3/30, 10%, *P* = 0.017). *Gardnerella vaginalis* was over‐represented in women presenting with symptoms (3/7, 43% versus 0/13, 0%, *P* = 0.03, linear discriminant analysis, LDA (log 10) and cases culminating in miscarriage (3/6, 50% versus 0/14, 0%, *P* = 0.017). In the majority of cases (10/14, 71%) bacterial composition was unchanged following cerclage insertion and perioperative interventions.

**Conclusions:**

Reduced relative abundance of *Lactobacillus* spp. is associated with premature cervical dilation, whereas high levels of G. vaginalis are associated with unsuccessful rescue cerclage cases. The insertion of a rescue cerclage does not affect the underlying bacterial composition in the majority of cases.

**Tweetable abstract:**

Preterm cervical dilatation associates with reduced *Lactobacillus* spp. Presence of *Gardnerella vaginalis* predicts rescue cerclage failure.

## Tweetable abstract

Preterm cervical dilatation associates with reduced *Lactobacillus* spp. Presence of *Gardnerella vaginalis* predicts rescue cerclage failure.

## Introduction

Preterm birth (PTB) is the leading cause of death among children under the age of 5 years, with many survivors suffering significant morbidity.^1^ Approximately 1% of women experience painless cervical dilation in the second trimester,^2^ which has been attributed to subclinical infection, recurrent vaginal bleeding or cervical insufficiency. Previous studies employing culture‐dependent techniques have identified strong associations between the presence of bacterial vaginosis and second trimester miscarriage.^3^ The majority of these cases also exhibit chorioamnionitis,^4^ highlighting the link between vaginal microbial community structure, ascending infection, and late miscarriage. Recently culture‐independent techniques have been used to examine the composition of vaginal microbiota in pregnancies delivering at term,^5^ preterm,^6^ prior to PPROM^7^ before and after history/ultrasound indicated cervical cerclage^8^ and following progesterone treatment.^9^ There is increasing evidence that a vaginal microbiome dominated by *Lactobacillus* species such as *Lactobacillus crispatus* is associated with healthy reproductive outcomes, whereas *Lactobacillus* spp. depletion or atypical vaginal colonisation has been associated with subsequent PTB^6^, ^8^ and PPROM.^7^ Management of women presenting with cervical dilation and exposed fetal membranes in the second trimester is challenging. If left untreated, most will deliver within 2–3 weeks, even if asymptomatic at presentation,^10^, ^11^, ^12^ resulting in miscarriage or extremely early preterm birth. The alternative is to insert an ‘emergency’ or ‘rescue’ cerclage. The optimal selection criteria, surgical technique, suture material, and perioperative management are unknown and rates of singleton delivery beyond 28 weeks’ gestation range from 40 to 80%.^13^, ^14^, ^15^, ^16^ Predicting which women will benefit from a rescue cerclage is difficult. It is accepted that cerclage is inappropriate for women with active bleeding or sepsis, and that cerclage performed for women with advanced dilation and significant membrane prolapse is unlikely to be successful. However, methods to identify women with premature cervical dilatation as a result of cervical insufficiency who are likely to do well following cerclage and those with cervical dilation as a result of subclinical inflammation resulting from host:microbe interactions are lacking. Elevated levels of acute phase proteins such as CRP appear to have some capacity to predict poor outcome^13^, ^14^ and more recently it was reported that positive aerobic culture of vaginal bacteria at the time of cerclage may predict subsequent preterm delivery.^17^ However, detailed examination of the relation between vaginal bacterial composition as determined with culture‐independent methods and rescue cerclage outcomes has not been done.

In this study, we analyse the composition of vaginal bacteria using next generation sequencing (NGS) techniques, before and after rescue cerclage, and correlate these data with clinical outcomes.

## Methods

Patient and public involvement sessions within our prematurity research framework identified a need for more research to identify the cause of infection in women undergoing rescue cervical cerclage, and the need for more accurate diagnostic techniques to identify those women at risk of subsequent infection, in whom a cerclage is more likely to fail.

Women with a singleton pregnancy with cervical dilation and exposed fetal membranes in the second trimester who were candidates for rescue cerclage, were prospectively recruited between January 2015 and 2017. Rescue cerclage was defined as a cerclage placed in the presence of a dilated cervix and exposed fetal membranes at or beyond the external cervical os. Rescue cerclage was only performed in the absence of significant contractions, heavy vaginal bleeding or signs of maternal infection (temperature >37.5°C/tachycardia >110 bpm/purulent discharge). Cervicovaginal fluid was sampled from the posterior fornix under direct visualisation at the beginning of the rescue cerclage procedure using a BBL CultureSwab MaxV Liquid Amies swab (Becton, Dickinson and Company, Winnersh, UK) before any antibiotic treatment or vaginal cleaning. All cerclages were inserted by the same operating team using a standardised technique. The cervix was grasped with sponge‐holding forceps and a Foley catheter inserted through the external os and inflated to hold the fetal membranes at the level of the internal os. The cervix was then closed with monofilament loop Nylon 1.0 (Ethilon, W728, Edinburgh, UK) placed circumferentially around the cervix and tied anteriorly as the Foley catheter was deflated and removed from the cervix. Postoperatively all patients received 24 h intravenous antibiotics (cefuroxime and metronidazole), 3 days of indomethacin 100 mg PR OD (PR, per rectum; OD, once a day) and Progesterone 400 mg OD until 34 weeks’ gestation. A second cervicovaginal fluid sample was obtained 10 days after stitch insertion during a routine follow‐up appointment for those women who remained pregnant (14/20). A matched cohort of women (*n* = 30) with a closed cervix who subsequently delivered at term were also sampled at a comparable time of gestation (20–22 weeks).

DNA was extracted from the vaginal swabs and integrity confirmed by PCR amplification as previously described.^5^ The V1‐V2 hypervariable regions were amplified for sequencing using forward and reverse fusion primers. The forward primer set (28f‐YM) consisted of a mixture of the following primers mixed at a 4:1:1:1 ratio; 28F‐Borrellia GAGTTTGATCCTGGCTTAG; 28F‐ Chlorflex GAATTTGATCTTGGTTCAG; 28F‐ Bifido GGGTTCGATTCTGGCTCAG; 28F GAGTTTGATCNTGGCTCAG. The reverse primer consisted of; 388R TGCTGCCTCCCGTAGGAGT.^18^

Sequencing was performed at RTL Genomics (Lubbock, TX, USA) using an Illumina MiSeq platform (Illumina Inc., Cambridge, UK). Resulting sequence data were analysed using the MiSeq SOP Pipeline of the mothur package.^19^ Sequence alignment was performed using the Silva bacterial database (www.arb-silva.de/), and classification was performed using the RDP (Ribosomal Database Project) database reference sequence files and the Wang method.^20^ The RDP MultiClassifier script was used for determination of operational taxonomic unit taxonomies (phylum to genus), and species‐level taxonomies were determined using USEARCH.^21^ To avoid sequencing bias, data were resampled and normalised to the lowest read count (*n *=* *9666).

Examination of differences between vaginal microbiota was performed at genera level in the Statistical Analysis of Metagenomic Profiles software package (stamp).^22^ Samples were classified into three groups according to Centroid Linkage Hierarchical Clustering Analysis (HCA) of bacterial genera using a clustering density threshold of 0.75 with the 25 most abundant genera displayed. Three separate clusters were identified and characterised on the basis of *Lactobacillus* spp. abundance into dominant (75–100% abundance), intermediate (50–75% abundance), and deplete (0–50% abundance) (Supporting Information Figure S1).

Maternal and neonatal metadata were collected from the paper case‐files and the electronic databases, Cerner Millennium® and Badgernet®. Significant neonatal morbidity was defined as the presence of any of the following: intraventricular haemorrhage, necrotising enterocolitis, patent ductus arteriosus, retinopathy of prematurity or chronic lung disease. ‘Successful rescue cerclage’ was defined as the birth of a neonate who was discharged from hospital without identifiable morbidity. ‘Unsuccessful cerclage’ was defined as resulting in miscarriage, intrauterine death, neonatal death or neonatal morbidity.

**Figure 1:**
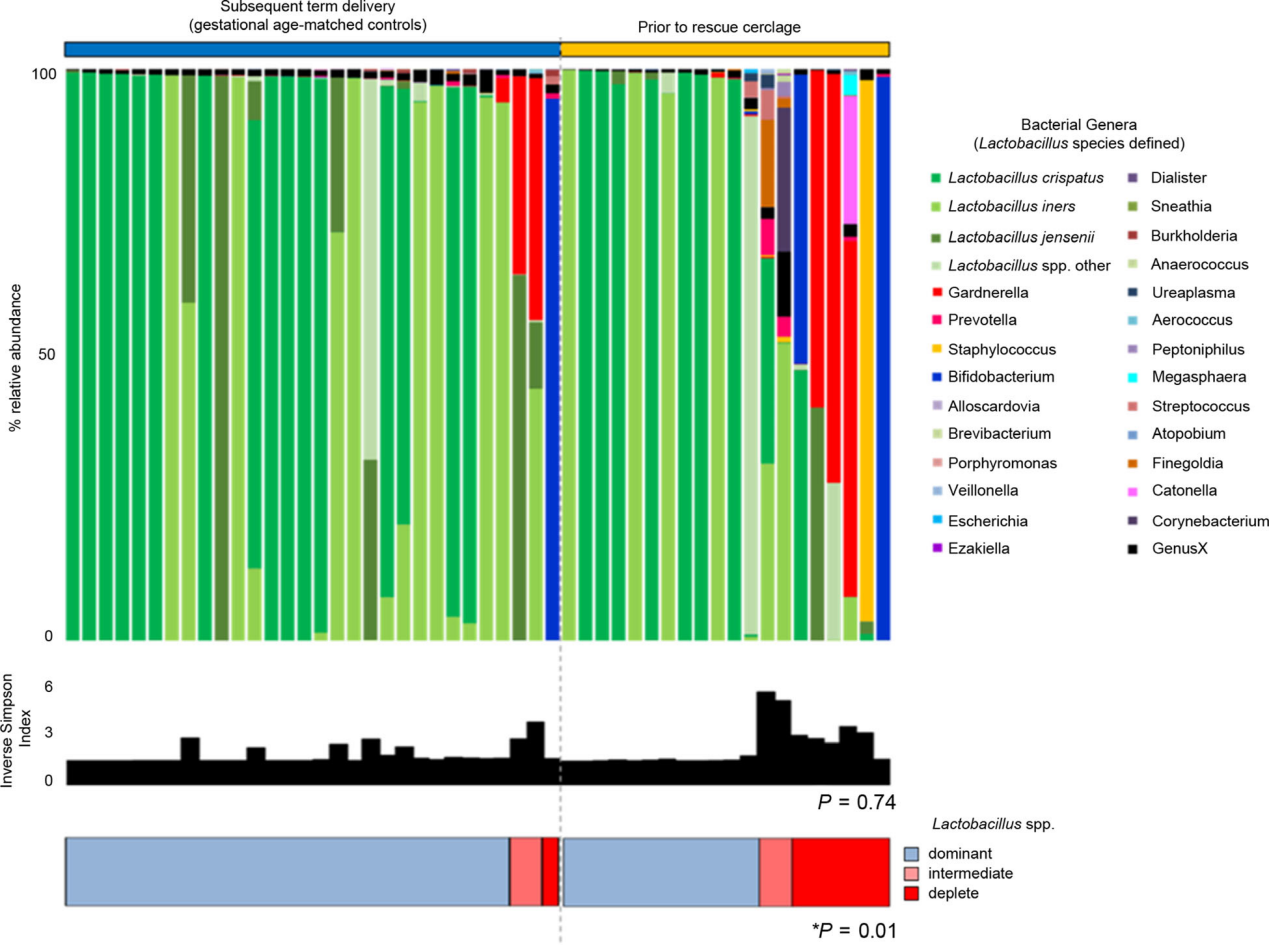
Vaginal microbial community structure prior to rescue cerclage compared with gestation age‐matched controls. Relative abundance of bacterial genera and *Lactobacillus* spp. (shades of green) within the vaginal microbiota, inverse Simpson index (diversity), and classification by *Lactobacillus* spp. dominance; dominant (blue) >75%, intermediate (pink) 50–75%, and deplete (red) <50%. Women with premature cervical dilation compared with gestation age‐matched controls (closed cervix subsequent term delivery). The proportion of women with a vaginal microbiome with reduced (intermediate or deplete) *Lactobacillus* spp. abundance was significantly higher (*P* = 0.01) in women prior to rescue cerclage.

**Figure 2:**
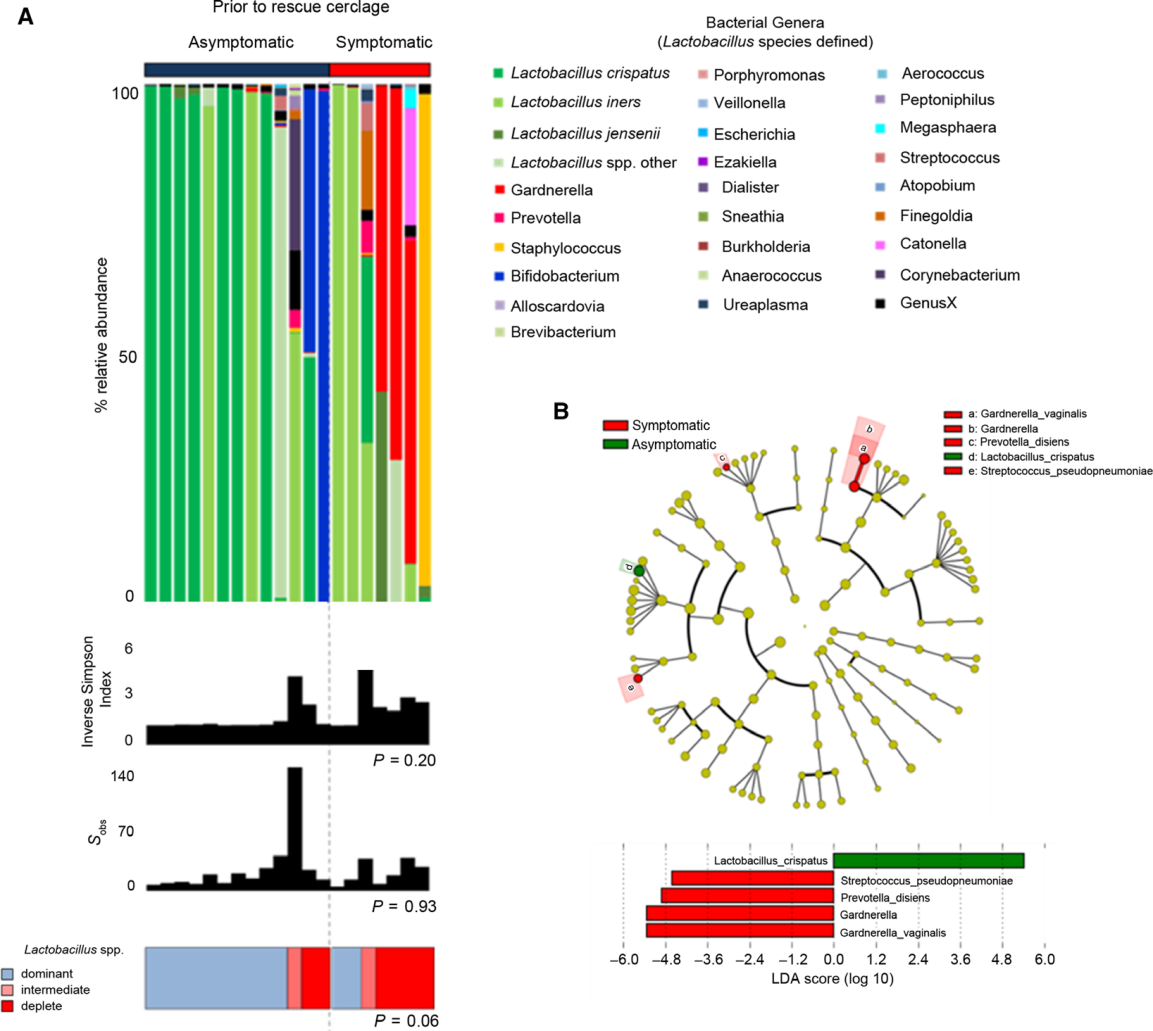
(A) Vaginal microbial community structure prior to rescue cerclage in women with and without symptoms. Relative abundance of bacterial genera and *Lactobacillus* spp. (shades of green) within the vaginal microbiome, inverse Simpson index (diversity), and classification by *Lactobacillus* spp. dominance; dominant (blue) >75%, intermediate (pink) 50–75%, and deplete (red) <50%. There were no significant differences in vaginal community structure between asymptomatic women and those presenting with symptoms. A difference at bacterial genera level within these communities was apparent with an overrepresentation of *Lactobacillus crispatus* (dark green) and *Bifidobacterium* (blue) in the asymptomatic cohort and *Gardnerella vaginalis* (red) in the symptomatic group. (B) *Gardnerella vaginalis* and *L. crispatus* are differentially expressed between women with and without symptoms prior to emergency cerclage. Cladogram describing differentially abundant vaginal microbial clades and nodes observed between asymptomatic women and those with symptoms as identified using LEfSe analysis. The effect size for each differentially abundant species was estimated using LDA. Vaginal microbiome of symptomatic women was enriched with *Gardnerella vaginalis*,* Prevotella disiens*, and *Streptococcus pseudopneumoniae,* whereas those without symptoms were comparatively enriched with *Lactobacillus crispatus*.

**Figure 3:**
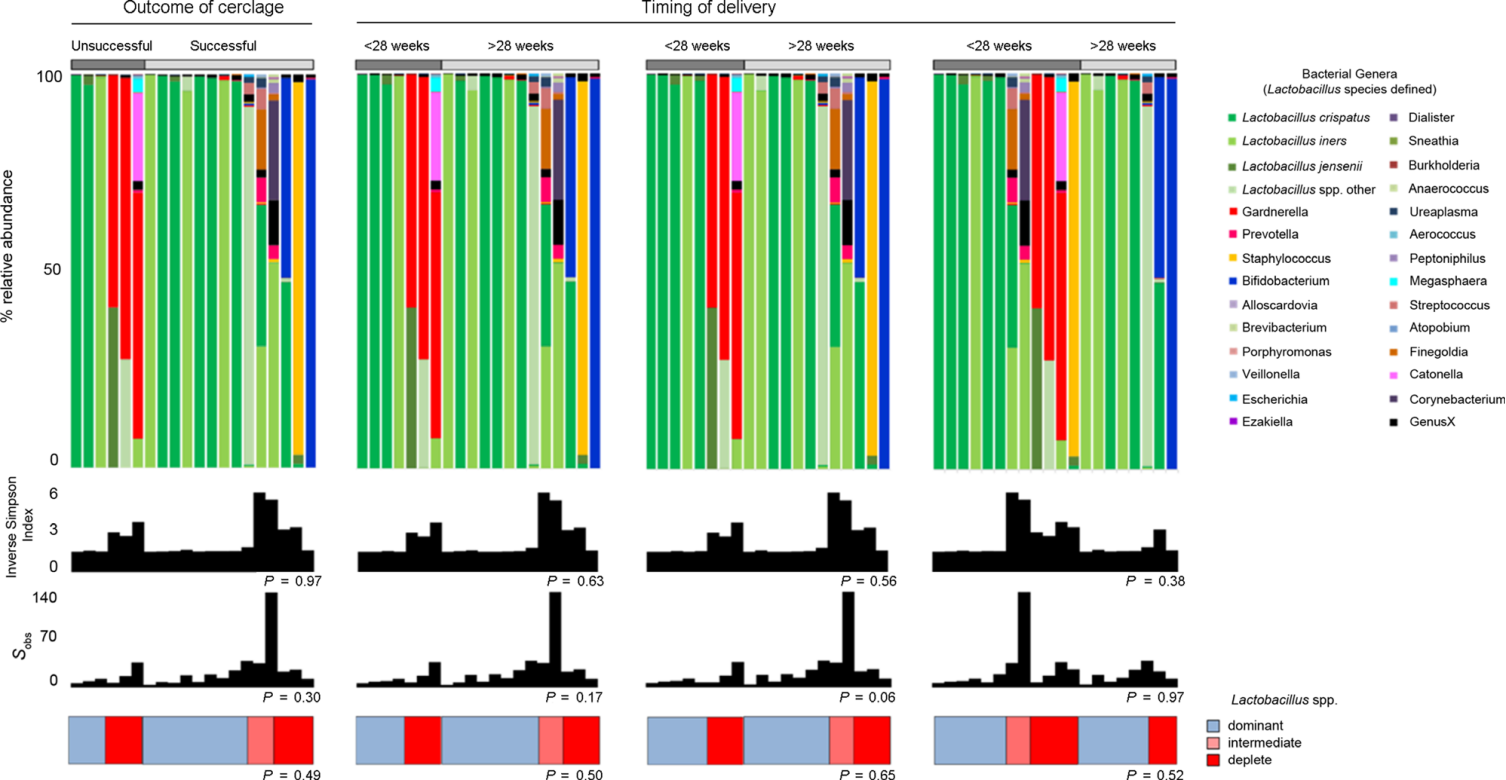
Vaginal microbial communities dominated by *Gardnerella vaginalis* were associated with poor outcomes. Relative abundance of bacterial genera and *Lactobacillus* spp. (shades of green) within the vaginal microbiome, inverse Simpson index (diversity), and classification by *Lactobacillus* spp. dominance; dominant (blue) >75%, intermediate (pink) 50–75%, and deplete (red) <50%. There were no significant differences in the proportion of women with a *Lactobacillus* spp.‐deplete microbiome between successful and unsuccessful cerclages or between cases delivering before and after 28, 32, and 37 weeks. The presence of *Gardnerella vaginalis* (red) was only seen prior to cerclages with unsuccessful outcomes.

**Figure 4:**
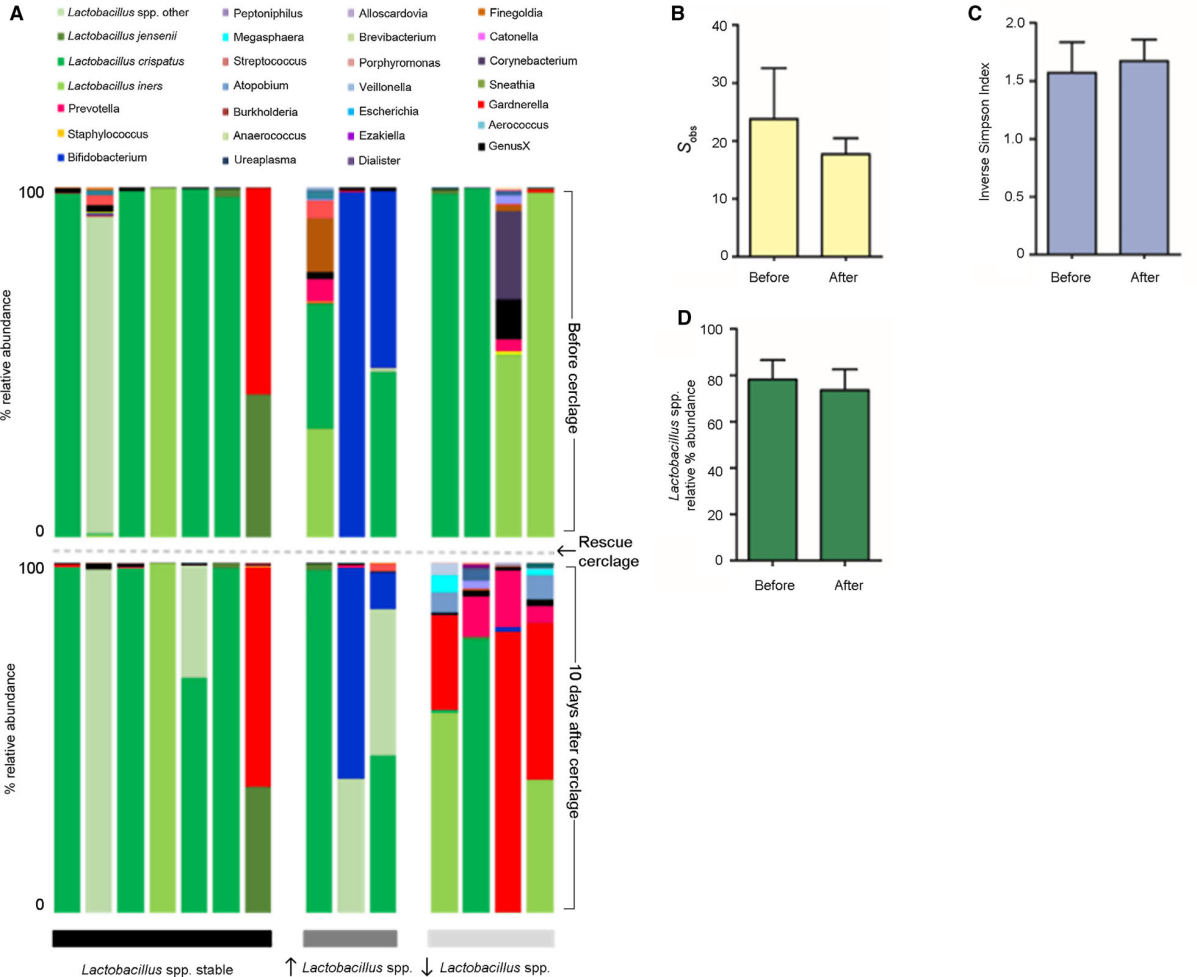
Rescue cerclage and postoperative interventions did not perturb the vaginal microbiome in the majority of cases. (A) Relative abundance of bacterial genera and *Lactobacillus* spp. (shades of green) within the vaginal microbiome before (top) and 10 days after (bottom) emergency cervical cerclage for cases where *Lactobacillus* spp. abundance remained stable (left, black), increased (middle, dark grey) and decreased (right, light grey). In 70% (10/14) of cases there was minimal change or increased *Lactobacillus* spp. abundance following rescue cervical cerclage. In the minority of cases (4/14, 30%) *Lactobacillus* spp. abundance fell and *Gardnerella vaginalis* emerged. Overall, no significant differences were observed in richness (B), diversity (C) or total *Lactobacillus* spp. abundance (D).

Vaginal bacterial composition from samples taken prior to rescue cerclage was compared with gestation age‐matched controls (Figure 1), and between rescue cerclage cases separated into the following groups: symptomatic versus asymptomatic women (Figure 2), high risk of PTB versus low risk (Figure S2), successful versus unsuccessful pregnancy outcome (Figure 3), before and after cerclage insertion (Figure 4), and cerclages resulting in delivery before and after 28, 32, and 37 weeks’ gestation (Figure 3).

The LEfSe method^23^ was used to identify differentially abundant taxonomic features between all groups. An *α* value of 0.05 was used for factorial Kruskal–Wallis test between classes with a minimum threshold of 2.0 used for logarithmic LDA scoring of discriminative features.

This work was supported by the Medical Research Council (grant MR/L009226/1), the Comprehensive Biomedical Research Centre at Imperial College London of the National Institute for Health Research (grant P45272), Imperial National Health Service Trust, and the Genesis Research Trust (grant P51389). Funders did not contribute to the conduct of the research or writing the manuscript.

## Results

A total of 20 women diagnosed with a dilated cervix and exposed fetal membranes were recruited and cervicovaginal samples taken prior to rescue cerclage. Fourteen were still pregnant 10 days later and were re‐sampled. Thirty women with uncomplicated pregnancy with closed cervices were recruited and sampled between 20 and 22 weeks [20^+4^ (20^+0^–22^+1^)]. Demographics including age, ethnicity, parity, and gestation at cerclage insertion were comparable between those women receiving cerclage and low‐risk controls (Table 1).

**Table 1:** Baseline characteristics of patients undergoing rescue cervical cerclage

Predictor of outcome (singleton)	Cervical dilation Exposed fetal membranes *n* = 20	Closed cervix uncomplicated term delivery *n* = 30	*P*‐value
**Age**	32 (29–34)	33 (31–36)	0.16
**Parity**			
Nulliparous	10 (50%)	21 (70%)	
Primiparous	4 (20%)	5 (17%)	
Multiparous	6 (30%)	4 (13%)	0.3
**Ethnicity**			
Black	9 (45%)	6 (20%)	
Asian	6 (30%)	9 (30%)	
Caucasian	5 (25%)	15 (50%)	0.11
**Risk factor**			
None	9 (45%)	30 (100%)	
Previous PTB	7 (35%)	0	
Previous MTL	4 (20%)	0	
Previous PTB + MTL	4	0	
LLETZ	0	0	
CS Full dilation	0	0	
**Gest. at cerclage insertion**	19^+4^ (18^+0^–22^+2^)	N/A	
**Gest. at microbiome sampling**	19^+4^ (18^+0^–22^+2^)	20^+4^ (20^+0^–22^+1^)	0.11
**Cervical dilation**	3 (1.25–3.0)	N/A	
≥2 cm	11 (55%)		
>2 cm	9 (45%)		
**Presence of symptoms**	7 (35%)	0	
**Markers of infection**			
CRP (Median)	13.3 (3.8–23.4)	N/A	
CRP (Mean)	14.0 (6.9–21.1)		
WCC (Median)	10.0 (8.2–13.0)		
WCC (Mean)	10.9 (8.7–13.0)		

CRP, C‐reactive protein; Gest, gestational age; LLETZ, large loop excision of the transformation zone; MTL, mid‐trimester loss; PTB, preterm birth; WCC, white cell count.

Data presented as number (percentage) or mean 95% CI. *P*‐values: Mann–Whitney, Fishers Exact test.

### Rescue cervical cerclage outcomes

Of the 20 prospectively recruited cases of rescue cerclage there were: 16 live births [the majority of which were beyond 28 weeks (13/16)], three miscarriages, one intrauterine death (IUD), and two neonatal deaths (NND), resulting in 14 babies discharged home from the neonatal unit without morbidity (Table 2). These data are comparable to outcomes from larger retrospective studies.^24^

**Table 2:** Outcomes following prospectively recruited cases of rescue cervical cerclage

Outcome	Rescue cervical cerclages (*n* = 20)
**Unsuccessful**	6 (30%)
PPROM sPTB 26/40 NND	1 (5%)
Chorioamnionitis 20/40 MTOP	1 (5%)
PPROM 18/40 MTOP	1 (5%)
PPROM 22/40 Miscarriage	1 (5%)
Intrauterine death @ 25/40	1 (5%)
sPTB 24/40 NND	1 (5%)
**Successful**	14 (70%)
Delivery	
25–27^+6^	1 (5%)
28–31^+6^	1 (5%)
32–36^+6^	5 (15%)
37+	7 (35%)
**Average gestational age at delivery**	35^+1^ (26^+3^–38^+5^)

IUD, intrauterine death; MTOP, medical termination of pregnancy; NND, neonatal death.

Data presented as number (percentage).

### Reduced *Lactobacillus* spp. abundance was associated with cervical dilation and exposed membranes

In comparison with gestation age‐matched controls, women with a dilated cervix and exposed fetal membranes sampled before cerclage were more likely to have low relative abundance or absence of *Lactobacillus* spp. [3/30 (10%) versus 8/20 (40%), *P* = 0.017], but overall microbial diversity was similar (*P* = 0.74) (Figure 1). The presence of PTB risk factors was not associated with vaginal microbiota composition (Figure S2). Patients presenting with symptoms such as increased vaginal discharge or mild discomfort prior to rescue cerclage were more likely to have reduced or absent *Lactobacillus* spp. [5/7 (71%) versus 3/13 (23%), *P* = 0.06] and the presence of *G. vaginalis* (3/7, 43% versus 0/13, 0%). LEfSe analysis of differentially abundant taxonomic features between symptomatic and asymptomatic patients identified *G. vaginalis*,* Prevotella disiens*, and *Streptococcus pseudopneumoniae* as discriminatory taxa for symptomatic patients; *L. crispatus* was associated with asymptomatic patients (Figure 3).

### *Gardnerella vaginalis* prior to rescue cerclage is associated with cerclage failure

The proportion of women with *Lactobacillus* spp.‐deplete microbiome a was similar between successful and unsuccessful cases (3/6, 50% versus 5/14, 36%, *P* = 0.64 Fisher's Exact test) and when compared across gestational age at delivery [<28 weeks, 3/6, 50% versus 5/14, 36%, *P* = 0.64), <32 weeks (3/8, 38% versus 5/12, 42%, *P* = 1.0) and <37 weeks (6/12, 50% versus 2/8, 25%, *P* = 0.37, Fisher's Exact test)] (Figure 4). *Gardnerella vaginalis* was only seen before emergency cerclages that ultimately failed (Supporting Information Figure S3) as a result of: (1) PTB with NND at 26/40, (2) chorioamnionitis leading to MTOP at 20/40, and (3) PPROM the day after cerclage resulting in MTOP in 18/40 (3/6, 50% versus 0/14, *P* = 0.017 Fisher's Exact) (Figure 4).

In women where cerclage was deemed successful but delivery occurred preterm (<37 weeks) half (6/12) had a microbiome that was *Lactobacillus* spp. Deplete, whereas all those that delivered at term (8/8) had a vaginal microbiome dominated by *Lactobacillus* or *Bifidobacteria* spp. prior to rescue cerclage.

### Insertion of a rescue cervical cerclage and postoperative interventions have minimal impact upon vaginal microbiota

*Lactobacillus* spp. abundance, diversity, and richness was unchanged by rescue cerclage and postoperative interventions (Figure 4). The majority of samples taken 10 days after cerclage following intravenous antibiotics, indomethacin, and progesterone demonstrated similar or increased *Lactobacillus* spp. levels (10/14, 71%). In the remaining four cases there was a fall in *Lactobacillus* spp. abundance with the emergence of *G. vaginalis* (3/4 cases). Of these three cases, two delivered preterm at 30^+2^ and 34^+2^ weeks, and the third delivered at term (38^+4 ^weeks).

## Discussion

### Main findings

Reduced *Lactobacillus* spp. abundance is associated with premature cervical dilation.

High relative abundance of *G. vaginalis* is associated with maternal symptoms prior to rescue cerclage and subsequent cerclage failure.

Insertion of a rescue cerclage in combination with intravenous antibiotics, PR progesterone, and indomethacin does not perturb the vaginal microbiome in the majority of cases.

### Strengths and limitations

Strengths include the consistent operative technique, cerclage material, and postoperative management employed for all cases. The use of primers capable of detecting all of the *Lactobacillus* spp. and a wide range of pathobionts including *G. vaginalis*, provides the first detailed description of vaginal bacterial composition.

Limitations include the absence of an expectantly managed group, with premature cervical dilation, in whom a cerclage was considered but not performed, and the relatively low sample size.

### Interpretation

The management of women who present with silent cervical dilation and exposed fetal membranes in the second trimester of pregnancy remains challenging and contentious. As with preterm labour in general, patients likely present with silent cervical dilation due to differing underlying aetiologies. There has only been one small randomised trial of 30 women,^10^ which indicated that rescue cerclage may be associated with a reduction in risk of PTB before 34 weeks and an improvement in neonatal survival. However, that study included unbalanced allocation of women with multiple pregnancy to treatment groups, despite evidence that cervical cerclage in multiple pregnancy is less effective and may increase the risk of PTB.^24^ The remainder of the evidence base for the use of rescue cerclage consists of cohort studies. A recent systematic review^25^ found that rescue cerclage increases neonatal survival and significantly prolongs pregnancy. A large cohort study reported by Pereira et al.^11^ showed a bimodal distribution for gestational age of delivery in women receiving rescue cerclage. Ehsanipoor and colleagues propose that this bimodal distribution is due to the underlying aetiology of cervical dilation and that there may be two subsets of women, those destined for favourable outcomes following cerclage and those likely to deliver early because of subclinical infection.^25^ We, and others,^9^ have demonstrated associations between the bacterial community structure of the vaginal microbiome, and the risk and outcome of spontaneous preterm labour^6^, ^9^ and PPROM.^7^ We have also shown that cervical cerclage is effective in preventing PTB in women with mechanical damage to their cervix caused by ablative treatment for cervical intraepithelial neoplasia.^7^, ^26^ We therefore hypothesised that women presenting with silent cervical dilation represent at least two aetiological groups. In one group, cervical dilation occurs because of untimely cervical remodelling driven by inflammation resulting from adverse host:microbe interactions in the cervicovaginal and/or lower uterine space. In the other group, cervical dilation is the result of mechanical weakness. It is this latter group which is more likely to respond favourably to rescue cerclage.

To test our hypothesis, we undertook a prospective cohort study to examine vaginal bacterial composition before and after rescue cerclage and its relation to pregnancy outcome. Our results indicate that women with a non‐*Lactobacillus* spp.‐dominant microbiome more likely to develop premature cervical dilation and high levels of *G. vaginalis* prior to cerclage, are associated with mild non‐specific symptoms such as vaginal discharge and poor pregnancy outcome encompassing PPROM, chorioamnionitis, and miscarriage.

*Gardnerella vaginalis* stimulates the production of interleukin (IL)‐1β, tumour necrosis factor (TNF)‐α, IL‐6, and Human β defensins 1/2/3 by choriodecidua *in vitro*.^27^ It has been found to colonise the vagina in 16% of PTB cases compared with 3% of term pregnancies,^28^ has previously been associated with PTB following emergency cerclage in culture‐dependent studies,^17^ and is associated with chorioamnionitis.^29^, ^30^ *Gardnerella vaginalis* also forms biofilms, making it resistant to antibiotic treatment, which may account for the poor outcomes in this study when the fetal membranes were exposed to the vagina despite antibiotic treatment.^31^

Interestingly, two of the control samples also contained *G. vaginalis*, albeit at a lower abundance. The presence of *G. vaginalis* in women delivering at term^32^ and in non‐pregnant women with asymptomatic bacterial vaginosis^33^ suggests variation in pathogenicity between *Gardnerella* strains. There are 15 strains of *G. vaginalis*,^34^ each with differing ability to form biofilms and produce enzymes such as sialidase and vaginolysin, capable of damaging epithelial surfaces, two of many factors that influence pathogenic potential.^34^, ^35^ Variation in host microbe interaction, overall bacterial load, and the presence or absence of cervix protecting the fetal membranes (from bacteria within the vagina) are also likely to influence the response of an individual to a particular pathobiont.

Conversely, *L. crispatus* dominance of vaginal microbial communities is associated with asymptomatic cases and term delivery. This identifies the potential clinical utility for a PCR‐based^8^ or mass spectrometry‐based method of bacterial detection permitting real time characterisation of vaginal microbiota in a clinical setting.^36^ This would enable obstetricians to identify a subset of women who may be at increased risk of premature cervical dilation and those most likely to experience ascending infection following rescue cerclage culminating in PPROM, chorioamnionitis, and miscarriage.

Our data also suggest that rescue cerclage and perioperative interventions (antibiotics, progesterone, and indomethacin) do not lead to major, consistent shifts in vaginal microbiota composition, at least within the time frame of the study. In all cases, the suture material used for the procedure was monofilament nylon, which we have previously shown to have no effect upon vaginal microbial communities, unlike the more commonly used braided Mersilene, which is associated with a shift towards *Lactobacillus* spp.‐deplete, high‐diversity vaginal microbial communities.^8^ In 70% (10/14) of cases, we observed either minimal change or increased *Lactobacillus* spp. abundance following rescue cerclage. This provides some reassurance that the insertion of a monofilament rescue cerclage does not promote adverse vaginal microbiota, which may inadvertently promote membrane rupture and chorioamnionitis, leading to a poor outcome.

## Conclusion

This study contributes to the growing body of evidence that vaginal colonisation by *L. crispatus* is associated with term pregnancy. An absence of *Lactobacillus* spp. is emerging as a risk factor for preterm birth including premature cervical dilatation and enrichment with *G. vaginalis* prior to rescue cerclage is associated with symptomatology and poor outcomes. New rapid diagnostic techniques to assess vaginal bacterial composition could be developed to triage women into groups at high and low risk of premature cervical dilation and research programmes are underway to ascertain whether the vaginal microbiome can be modified in early pregnancy to promote *L. crispatus*. Identifying those women colonised with *G. vaginalis* prior to rescue cerclage (who may be more likely to develop ascending infection) provides additional prognostic information that can be used to manage patient expectations and provide enhanced postoperative surveillance.

### Disclosure of interests

PRB serves as a consultant for ObsEva, a company that works in the field of preterm birth. All other authors declare that they have no competing interests. Completed disclosure of interests forms are available to view online as supporting information. RB is supported by Imperial College National Health Service Trust, PRB is supported by the UK National Institute for Health Research Biomedical Research Centre (grant P45272), based at Imperial College Healthcare National Health Service Trust and Imperial College London. DAM is supported by the Medical Research Council (grant MR/L009226/1). Completed disclosure of interests form available to view online as supporting information.

### Contribution to authorship

RGB, PRB, and DAM conceived and designed the study. Patient recruitment and sample collection were undertaken by RGB, DC, and VT. Experiments were performed by RGB, DC, and YSL. Data analyses and interpretation were performed by RGB, DC, VT, JRM, AS, DAM, and PRB. All figures and tables were generated by RGB. The manuscript was written by RGB, PRB, and DAM and critically reviewed by all authors. All authors read and approved the final manuscript.

### Details of ethics approval

The prospective arm of this study was approved by National Health Service (NHS) National Research Ethics Service Committee London–Stanmore (REC 14/LO/0328) 26/03/2014.

### Funding

This work was supported by the Medical Research Council (grant MR/L009226/1), the Comprehensive Biomedical Research Centre at Imperial College London of the National Institute for Health Research (grant P45272), Imperial National Health Service Trust, and the Genesis Research Trust (grant P51389).

### Data availability

Public access to sequence data sets generated in this study along with accompanying metadata can be obtained from the Sequence Read Archive of the European Nucleotide Archive (Source number pending please contact authors for details).

## Supporting information


**Figure S1.** Hierarchical clustering analysis of the 25 most abundant bacteria genera in samples taken before rescue cerclage and from gestation age‐matched controls.
**Figure S2.** Vaginal microbial community structure prior to rescue cerclage in women at low and high risk.
**Figure S3.** Vaginal microbial community structure in cases of unsuccessful rescue cerclage.
