# Supplementary material for: Prospective observational study of vaginal microbiota pre‐ and post‐rescue cervical cerclage
Source: BJOG. 2019 Mar 10;126(7):916–25. doi: 10.1111/1471-0528.15600 (PMC6750128; doi:10.1111/1471-0528.15600)
Supplement: Supplementary file 1 — Figure S1. Hierarchical clustering analysis of the 25 most abundant bacteria genera in samples taken before rescue cerclage and from gestation age‐matched controls. Figure S2. Vaginal microbial community structure prior to rescue cerclage in women at low and high risk. Figure S3. Vaginal microbial community structure in cases of unsuccessful rescue cerclage. [file BJO-126-916-s001.pdf]

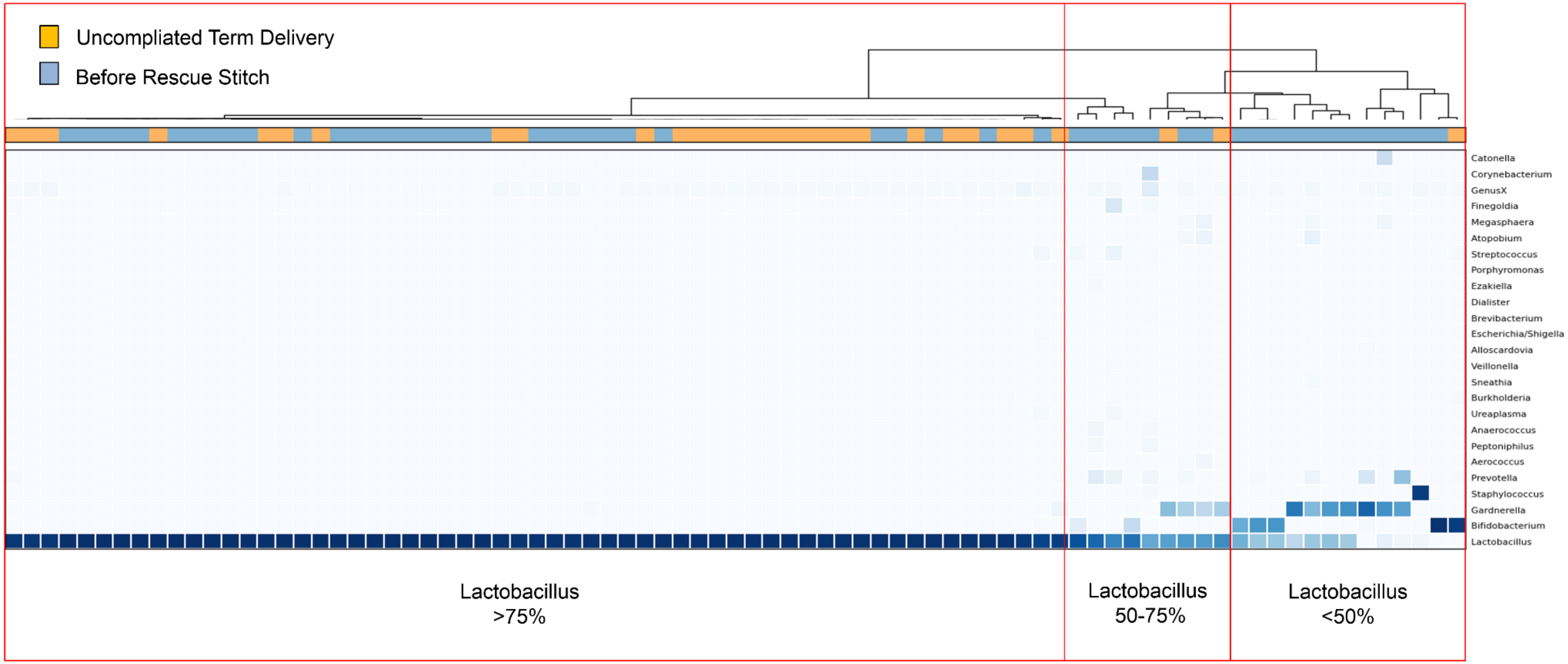

Figure S1. Hierarchical clustering analysis of the 25 most abundant bacteria genera in samples taken before rescue cerclage and from gestational age matched controls. Samples cluster into three groups defined by the abundance of *Lactobacillus* spp. dominant (>75%), intermediate (50-75%) and deplete (<50%).

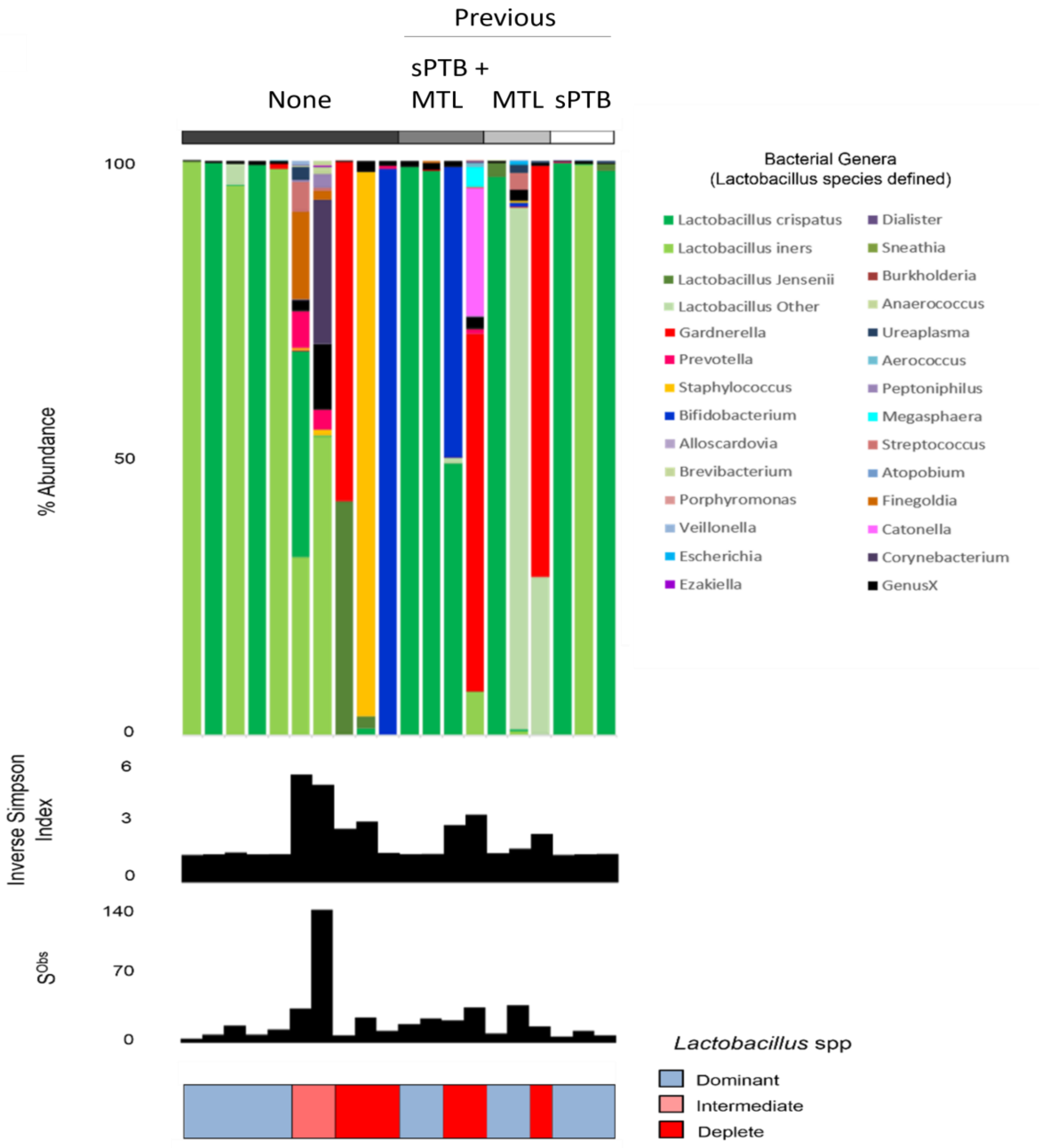

Figure S2. Vaginal microbial community structure prior to rescue cerclage in women at low and high risk. Relative abundance of bacterial genera and *Lactobacillus* spp. (shades of green) within the vaginal microbiome, inverse Simpson index (diversity) and classification by *Lactobacillus* spp. dominance; dominant (blue) >75%, intermediate (pink) 50-75% and deplete (red) <50%. There were no significant differences in vaginal microbial composition between women with and without pre-existing risk factors.

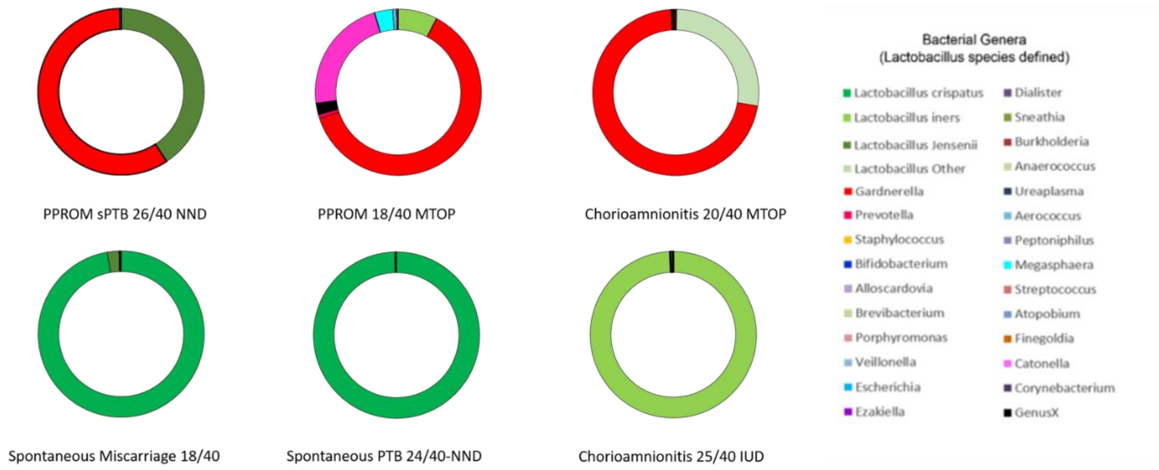

Figure S3. Vaginal microbial community structure in cases of unsuccessful Rescue cerclage. (A) Relative abundance of bacterial genera and *Lactobacillus* spp. (shades of green) within the vaginal microbiome for all 6 cases of unsuccessful rescue cerclage.
